# Spatio-temporal risk prediction of leptospirosis: A machine-learning-based approach

**DOI:** 10.1371/journal.pntd.0012755

**Published:** 2025-01-16

**Authors:** Rodrigue Govan, Romane Scherrer, Baptiste Fougeron, Christine Laporte-Magoni, Roman Thibeaux, Pierre Genthon, Philippe Fournier-Viger, Cyrille Goarant, Nazha Selmaoui-Folcher

**Affiliations:** 1 Institute of Exact and Applied Sciences, University of New Caledonia, Nouméa, Province Sud, New Caledonia; 2 Sciences and Technology Department, University of New Caledonia, Nouméa, Province Sud, New Caledonia; 3 Pasteur Institute of New Caledonia, Nouméa, New Caledonia; 4 HydroSciences Montpellier, University of Montpellier, CNRS, IRD, Nouméa, New Caledonia; 5 College of Computer Science and Software Engineering, Shenzhen University, Nanshan District, Shenzhen, China; 6 Public Health Division, The South Pacific Community, Nouméa, Province Sud, New Caledonia; University of Connecticut Health, UNITED STATES OF AMERICA

## Abstract

**Background:**

Leptospirosis is a neglected zoonotic disease prevalent worldwide, particularly in tropical regions experiencing frequent rainfall and severe cyclones, which are further aggravated by climate change. This bacterial zoonosis, caused by the *Leptospira* genus, can be transmitted through contaminated water and soil. The Pacific islands bear a high burden of leptospirosis, making it crucial to identify key factors influencing its distribution. Understanding these factors is vital for developing targeted policy decisions to mitigate the spread of *Leptospira*.

**Methodology/Principal findings:**

This study aims to establish a precise spatio-temporal risk map of leptospirosis at a national scale, using binarized incidence rates as the variable to predict. The spatial analysis was conducted at a finer resolution than the city level, while the temporal analysis was performed on a monthly basis from 2011 to 2022. Our approach utilized a comprehensive strategy combining machine learning models trained on binarized incidences, along with descriptive techniques for identifying key factors. The analysis encompasses a broad spectrum of variables, including meteorological, topographic, and socio-demographic factors. The strategy achieved a concordance metric of 83.29%, indicating a strong ability to predict the presence of contamination risk, with a sensitivity of 83.93%. Key findings included the identification of seasonal patterns, such as the impact of the El Niño Southern Oscillation, and the determination that rainfall and humidity with a one-month lag are significant contributors to *Leptospira* contamination. Conversely, soil types rich in organic matter may reduce bacterial presence and survival.

**Conclusions/Significance:**

The study highlights the significant influence of environmental factors on the seasonal spread of *Leptospira*, particularly in tropical and subtropical regions. These findings are crucial for public health planning, providing insights for targeted policies to reduce leptospirosis, while advanced machine learning models serve as a robust tool for improving disease surveillance, and risk assessment, which ultimately supports the development of an early warning system.

## Author summary

Leptospirosis is a neglected worldwide zoonosis caused by the *Leptospira* bacteria, responsible for nearly 60,000 deaths annually. It is transmitted to humans through contact with infected mammals and contaminated environments, where mammals, particularly rodents, spread the bacteria through their urine. Climate change, which increases heavy rainfall and severe cyclones, further exposes tropical and subtropical regions to bacterial contamination. Additionally, in the South Pacific, the specificities of the local environment and the lifestyle of the inhabitants contribute to increase exposure to the bacteria. In this paper, we conducted a precise spatio-temporal analysis of the risk of *Leptospira* contamination, incorporating environmental and socio-demographic factors in a subtropical region (New Caledonia) over a 12-year period. This study will facilitate the implementation of timely, targeted prevention and mitigation actions, benefiting public health services. Ultimately, the analysis revealed that accumulated rainfall and humidity with a 1-month lag, as well as the soil types specific to New Caledonia, are the primary factors contributing to the risk of *Leptospira* contamination.

## Introduction

Leptospirosis is a worldwide bacterial disease caused by pathogenic spirochetes of the genus *Leptospira* and is considered a zoonosis. Responsible for over a million cases annually throughout the world with nearly 60,000 deaths each year [1], the disease remains significantly neglected [2]. More specifically, the average global case fatality rate is estimated at 6.85%, but this rate can reach up to 30% in certain developing countries [1]. The majority of leptospirosis cases and deaths occur in tropical regions, with 73% of global cases and fatalities reported in areas between the Tropics of Cancer and Capricorn [1]. Although *Leptospira* can live in the kidney tubules of all mammals, rodents remain the main reservoir by dispersing the bacteria through their urine in soils and waters. Additionally, the bacteria can survive in the environment for months [3], leading to human infections through wounds or mucous membranes after exposure to an environment contaminated by infected animals, more often than through direct contact with the animals themselves. Humans are considered to be an incidental and dead-end host in the transmission chain, as human-to-human transmission is extremely rare [4]. Infections can lead to several complications, such as kidney and lung failure, and potentially Weil’s disease [5].

The incidence of the disease is influenced by numerous factors, notably environmental ones such as heavy rainfall [6], leading to a lower incidence rate in temperate regions compared to tropical and subtropical regions. This is particularly notable in developing countries where climate promotes the survival of *Leptospira* [7]. Indeed, it has been demonstrated that the pathogenic *Leptospira* present in the soil and freshwater sediments are brought to the surface water by heavy rainfall [6, 8], which increases the contamination risk and can lead to human infections, especially in tropical and subtropical regions [3] that experience heavy rainfall [8]. Additionally, the lifestyle in these areas favors human contact with the environment [9]. Besides the rainfall variable, temperature, altitude and soil types are the primary factors explaining the leptospirosis distribution, as climatic events facilitate the dispersion of *Leptospira* within the environment [10–12].

Recent works intended to establish a spatial and temporal analysis of the leptospirosis risk. For instance, in South Brazil [13], researchers forecasted the incidence rate of leptospirosis over a 12-year period in order to identify the temporal trends and high-risk areas for *Leptospira* transmission as the southern region shows the highest morbidity and mortality rates in the country. Using time series forecasting models such as the Seasonal AutoRegressive Integrated Moving Average (SARIMA) model, their findings highlighted the seasonal trend of incidence rates, revealing a seasonal pattern with a higher incidence rate predicted during the warm season. However, despite the promising results, the authors assumed that the incidence in a given month can be predicted solely based on previous incidence rates, with the seasonal pattern being the only variable considered. In addition to the seasonal pattern identification, our strategy aims to determine key factors in the risk of *Leptospira* contamination.

In Southeast Asia, a study was conducted to determine the environmental factors that may explain the distribution of leptospirosis incidence [14]. Although the authors highlighted the importance of the variance of slope, the wettest quarter, and the hottest quarter in the incidence predictions, these factors were identified based on incidences of leptospirosis in Thailand from 2013 to 2019, which did not allow to determine precisely the seasonal pattern of leptospirosis outbreaks. Therefore, this time scale did not take into account the seasonal pattern of leptospirosis outbreaks, which occur during the rainy season, as has been proven [15].

In the South Pacific, a study has been conducted in the Fiji Islands [16], including multiple factors such as rainfall, land cover and poverty rate. Indeed, following the leptospirosis outbreak that occurred in 2012, the authors proposed a cross-sectional seroprevalence study. As a result, they reported that 19.5% of the participants had antibodies indicative of either past or recent leptospirosis infection. In addition, using a multivariate logistic regression analysis, they identified heavy rainfall and proximity to water sources as crucial roles in disease transmission. Although the authors based their analysis on questionnaires and geographic information systems data, they analyzed the leptospirosis outbreak that occurred during the year 2012 alone. Therefore, the temporal dynamics has not been integrated to evaluate the seasonal pattern as we aimed to achieve in this paper.

In New Caledonia, only a few studies have been conducted to determine risk factors of *Leptospira* contamination in the country [17, 18]. However, the temporal dynamics involving the seasonal pattern has not been properly integrated. For example, the warm and rainy environment in New Caledonia increases leptospirosis outbreaks, particularly during La Niña periods of El Niño Southern Oscillation (ENSO) [18]. In addition, the population in New Caledonia lives according to three main lifestyles: urban, rural and tribal i.e., indigenous communities with a traditional lifestyle. Over 20% of the population live in tribes [19] and rely on fishing, hunting and subsistence agriculture, which may increase the interaction with the environment and therefore, their exposure to *Leptospira* [20, 21]. With the rainfall and tropical cyclone episodes getting more frequent and severe due to climate change [22], zoonotic diseases such as leptospirosis will occur more frequently in tropical regions as New Caledonia [18]. Additionally, in New Caledonia, there has been a resurgence of the disease with over 1,000 cases in the last decade, particularly during the 2020–2022 period with almost 600 leptospirosis cases. A fine-scale spatio-temporal understanding of *Leptospira* contamination risk in New Caledonia would enable the implementation of timely and targeted prevention and mitigation actions.

To our knowledge, there has been no comprehensive examination of the spatio-temporal aspect of leptospirosis risk in a subtropical region, considering a broad spectrum of environmental and demographic factors. In this study, we aim to establish a risk mapping based on the locations of leptospirosis cases.

The objectives of this work are to (1) establish a risk mapping of leptospirosis in all regions of an archipelago in the South Pacific by integrating various factors using predictive models and (2) identify which factors contribute the most to the predicted risk using a descriptive approach. To conduct our study, we collected spatio-temporal data of all reported leptospirosis cases from 2011 to 2022 together with various environment data.

To establish this risk mapping, an Ensemble Learning approach including several machine learning models and an under-sampling technique was developed. The prevailing factors were then determined according to the model predictions. The spatial aspect was considered by computing incidence rates on an IRIS unit scale, which is an infra-municipal division that we are detailing in the next section. Finally, the temporal aspect has been taken into account by computing incidence rates per IRIS unit and considering environmental and socio-demographic variables on a monthly scale.

## Methods

### Study location

**Fig 1:**
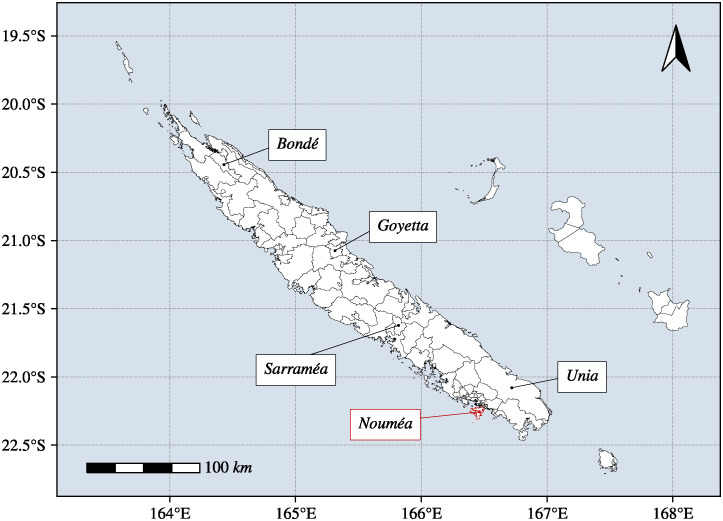
The study location is New Caledonia, divided into 114 IRIS (polygons). Initially, the archipelago had 162 IRIS, but those within the capital, *Nouméa*, were merged to minimize the number of imprecise location cases in the city. The IRIS border shapes were provided by the Institute of Statistics and Economic Studies of New Caledonia (https://ncl.popgis.spc.int/).

The spatial analysis has been conducted in the country of New Caledonia (Fig 1). With its 271,407 inhabitants divided into 33 cities on a total surface area of 18,576 *km*^2^ [25], the country is an archipelago composed of a main island called *La Grande Terre* and additional islands called *Les Iles Loyautés*. The climate of New Caledonia is subtropical with two major seasons: the warm and rainy season from November to April and the cold and dry season from May to October [26]. The archipelago was selected as study location due to its globally high leptospirosis incidence rate (97.27 cases per 100,000 inhabitants in 2022), diverse environmental conditions such as climate change, and availability of fine-grained epidemiological and environmental data, making it an ideal setting to study the spatial and temporal dynamics of the disease. Given the significant population discrepancy in New Caledonia, where its capital *Nouméa* accounts for 35% of the total population [25], this study examines the risk mapping of leptospirosis at the residential IRIS (Aggregated Units for Statistical Information) scale. This IRIS division has been developed by the French National Institute of Statistics and Economic Studies (INSEE) with the aim of having homogeneous units in terms of number of inhabitants. The IRIS division allowed to divide cities into several residential IRIS units where populations generally fall between 1,800 and 5,000 inhabitants. This unit division is homogeneous in terms of living environment and the boundaries of the unit are based on the major dividing lines provided by the urban fabric (main roads, railways, bodies of water, etc.). Developed in 2008, the demographic characteristics of certain IRIS have evolved, although their geographic boundaries have not been updated by the institute in order to preserve continuity in the data publication series.

All of the archipelago of New Caledonia is composed of 162 IRIS described by polygons (Fig 1).

### Leptospirosis cases and ethics statement

**Fig 2:**
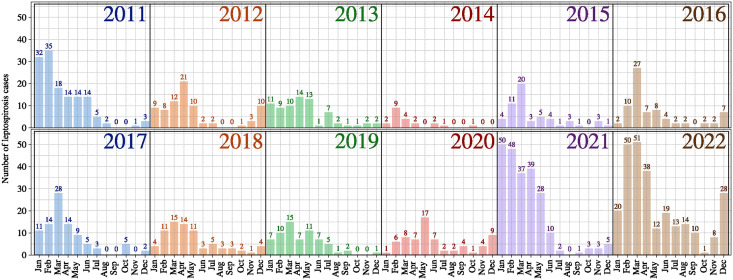
Total number of leptospirosis cases per month registered in New Caledonia.

For the purpose of this study, the Health and Social Affairs Department of New Caledonia provided individual cases of leptospirosis from 2011 to 2022 (Fig 2).

In New Caledonia, leptospirosis is a mandatory notifiable disease, and the following steps are involved. When a visit to a healthcare practitioner raises suspicion of leptospirosis, the doctor collects the patient’s information, symptoms, and epidemiological data using a standardized notification form. This form is then forwarded either to the public health service of the Health and Social Affairs Department of New Caledonia or to the central hospital laboratory, where diagnostic biological tests are conducted before the results are sent to the public health service. The form must include all relevant information regarding possible contamination within two weeks before symptom onset, any recreational activities or contact with animals (with the location) in the past 30 days, the symptoms experienced, and the diagnosis.

To preserve the anonymity of those involved, all reported cases have been anonymized by the organization so that no name, address, age or other information can be personally identifiable.

Most of the reported cases include the presumptive location, at the scale of a neighborhood or a tribe. By this available location, we associated each leptospirosis case to the corresponding IRIS. The reported cases also include the month and year of the infection which allowed us to compute the monthly incidence rate per 10,000 inhabitants according to population censuses. Since we were unable to locate most of the individual cases diagnosed in the capital of New Caledonia (*Nouméa*), we merged every IRIS within *Nouméa* to minimize the number of ignored cases in the area, i.e., leptospirosis cases with an imprecise location. By merging these IRIS (Fig 1), the number of units has been reduced from 162 to 114. In this study, the predictive risk mapping of leptospirosis was performed on a monthly basis, aligning with the registration of individual cases that occur each month. Thus, a total of 16,416 units (month-IRIS) were analyzed (114 IRIS × 12 months × 12 years) for the entire time-frame.

During the 2011–2022 period, New Caledonia has registered over 1,000 leptospirosis cases. Throughout this duration, the monthly registered incidence rates varied between 0 and 93.98 cases per 10,000 inhabitants, with the highest rate observed in the IRIS of *Sarraméa* (Fig 1) in January 2021. In this study, we aimed to develop a risk map of leptospirosis in New Caledonia based on the registered cases. Therefore, we categorized each incidence rate into two groups: an “absence of risk” group comprising all month-IRIS with a zero incidence rate, and a “presence of risk” group, which includes all the others. Even though binarizing incidence rates into “absence of risk” and “presence of risk’ removes the information about the number of reported cases, we considered that as soon as a leptospirosis case is recorded, the risk of contamination is present. Thus, a spatio-temporal analysis that predicts the absence or presence of contamination risk in a given month-IRIS seemed much more relevant to us, as the probability of the “presence of risk” (%) predicted from our strategy is actually a quantification whether the risk is high or not.

While our strategy is adjusted on the binarized month-IRIS incidence rates for each month between 2011 and 2020, we performed predictions on the binarized month-IRIS incidence rates for each month in 2021 and 2022. For a given month *m*, our models are trained to predict the contamination risk based on variables from previous months (*m* − 1, *m* − 2, and *m* − 3) which enable the prediction of contamination risk one month in advance.

### Data retrieval and preprocessing

Recent works have demonstrated the contribution of various factors in the *Leptospira* transmission, in particular landscape, topographic, meteorologic and demographic variables [10, 12, 27, 28]. Thus, to conduct a spatio-temporal analysis of leptospirosis in New Caledonia, we retrieved a wide range of variables that we are detailing in the next subsections.

#### Meteorologic variables

Meteorologic variables were measured according to the World Meteorological Organization (WMO) standards and provided with no gap by *Météo-France* of New Caledonia (Table 1). In New Caledonia, there are around 50 stations that measure rainfall, temperature and humidity. However, these stations do not fully cover the entire archipelago (i.e., not every IRIS has at least one installed weather station). Therefore, we employed an interpolation method to generate comprehensive raster maps with a resolution of 250 meters for the collected meteorological variables. These data were interpolated at the scale of the whole country using the AURELHY (Analysis Using the Topography for Hydrometeorology) method [29] which also complies to WMO standards. The interpolation process has been carried out using R language (version 4.0.0) and the following packages: aurelhy [30], sf [31], raster [32], terra [33], gstat [34] and automap [35]. The AURELHY interpolation method generates a representative raster map from point data, such as average temperature, using a binary mask and a landscape descriptor like a digital terrain model (DTM), also known as the altitude. The process begins by creating an initial empty raster by downscaling the DTM to a 250-meter resolution, which serves as the basis for interpolation. We set the final resolution to 250 meters in order to obtain a sufficiently precise representative map without significantly slowing down too much the process. For reference, starting with a 10-meter resolution altitude raster and 50 meteorological data points covering New Caledonia, we obtained a 250-meter resolution raster in about a week and a half. AURELHY then constructs a landscape matrix where each row represents a raster pixel and each column includes altitude and lagged altitude values from 6 distances and 8 angles. As a result, the lagged altitude values are composed of 6 *distances* × 8 *angles* = 48 values. Considering the altitude on the geographic position of a pixel and its 48 lagged altitude values, we have a total of 49 values to define the spatial relationship on a given pixel. Then, AURELHY applies a principal component analysis (PCA) to reduce these 49 variables to 10 principal components, simplifying the data dimension. The final step involves interpolating the meteorological variable using kriging on the retained principal components and any additional variables, such as the distance from the sea, to refine the interpolation and generate predictions for all pixels. To measure the quality of the interpolation, the correlation score (*R*^2^) can be computed on the adjusted kriging model. In our tests, the interpolated rainfall, temperature and humidity are correlated to the altitude and the distance from the sea with correlations equal to 0.5 ± 0.14, 0.76 ± 0.19 and 0.81 ± 0.13, respectively.

**Table 1:** Description of the variables retrieved and used in this work.

Variables (units)	Source
Rainfall	Weather stations measured by *Météo-France* of New Caledonia and provided by the French Government (https://meteo.data.gouv.fr/).
Average accumulated rainfall on month *m* − 1 (*mm*)	
Median of accumulated rainfall on month *m* − 1 (*mm*)	
Variance of accumulated rainfall on month *m* − 1	
Average accumulated rainfall on month *m* − 2 (*mm*)	
Median of accumulated rainfall on month *m* − 2 (*mm*)	
Variance of accumulated rainfall on month *m* − 2	
Average accumulated rainfall on month *m* − 3 (*mm*)	
Median of accumulated rainfall on month *m* − 3 (*mm*)	
Variance of accumulated rainfall on month *m* − 3	
Temperature	
Minimum of monthly temperature on month *m* − 1 (°C)	
Average of monthly temperature on month *m* − 1 (°C)	
Maximum of monthly temperature on month *m* − 1 (°C)	
Range of temperature $(T_{\text{max}}^{\circ }$ − $T_{\text{min}}^{\circ })$ on month *m* − 1 (°C)	
Humidity	
Average of monthly humidity on month *m* − 1 (%)	
Topography	Raster data of altitude with a resolution of 10 meters, provided by the Government of New Caledonia (https://georep.nc/).
Average of the altitude within an IRIS (*m*)	
Median of the altitude within an IRIS (*m*)	
Variance of the altitude within an IRIS	
Soil Type	Polygon data provided by the French National Research Institute for Sustainable Development [37].
20 categories (% of an IRIS covered by each category)	
Land Use	Land Use polygon data provided by the Government of New Caledonia (https://georep.nc/).
22 categories (% of an IRIS covered by each category)	
Farming area	Polygon data provided by the Environment Observatory of New Caledonia (https://oeil.nc/geoportail).
Proportion of an IRIS covered by a farming area (%)	
Fires	Point data provided by the Environment Observatory of New Caledonia (https://oeil.nc/geoportail).
Number of fires occurred on month *m* − 1 (occurrences)	
Demography	Polygon data provided by the Institute of Statistics and Economic Studies of New Caledonia (https://ncl.popgis.spc.int/).
Population within an IRIS	
Population density within an IRIS (*sq. km*)	
Number of people whose main mode of transportation is walking	
Number of people whose main mode of transportation is public transit	
Number of people living in tribes	
Number of people working in agriculture	
Number of households without individual access to water	
Number of households without access to electricity	
Number of houses where the construction ended at least 10 years ago	
Number of people working in a city other than their home city	
Number of stay-at-home people	
Number of people in the working class	

**Fig 3:**
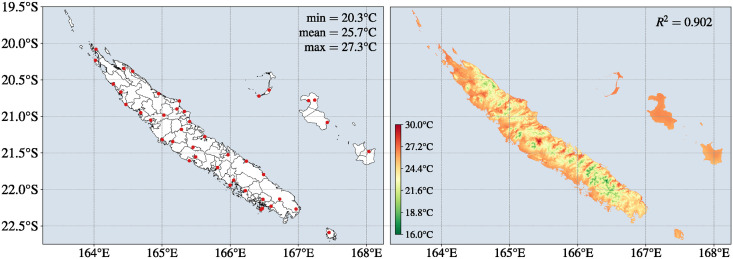
Mapping of the weather stations (red dots) in New Caledonia (left panel) and an example of an interpolated raster map obtained by AURELHY method (right panel). The interpolated map has been obtained by integrating the average temperature of December 2022, the altitude and the distance from the sea, resulting in a correlation *R*^2^ = 0.902. The IRIS border shapes and meteorological point data in the left panel were provided by the Institute of Statistics and Economic Studies of New Caledonia (https://ncl.popgis.spc.int/), and the *Météo-France* of New Caledonia (https://meteo.data.gouv.fr/), respectively.

Meteorologic factors are composed of accumulated rainfall (*mm*), average humidity (%), minimum temperature (°C), average temperature (°C), and maximum temperature (°C). We retrieved these factors for each month during the period from 2011 to 2022 and applied AURELHY method to obtain monthly representative maps during this period (Fig 3).

Once we computed the meteorological maps, it became necessary to extract the variables contained within each IRIS to form our dataset. This extraction process used a masking technique to ensure that only meteorological data (represented by pixels) geographically located within each IRIS were considered. Then, these variables have been integrated into the analysis by computing their mean, median, and variance within each IRIS. This integration was performed for an incidence rate computed on a given month *m* and took into account accumulated rainfall, temperatures (minimum, maximum and mean) and average humidity from the previous month *m* − 1. Moreover, we included the accumulated rainfall from the months *m* − 2 and *m* − 3 as two new variables, considering their impact on *Leptospira* resuspension [6]. Additionally, the monthly temperature range $(T_{\text{max}}^{\circ }$ − $T_{\text{min}}^{\circ })$ within each IRIS has also been computed and included into the analysis as one new variable.

#### Topographic and landscape variables

Topographic and landscape variables were provided by the Government of New Caledonia, the French National Research Institute for Sustainable Development, and the Environmental Observatory of New Caledonia (Table 1). The soil type variable, described by polygons, is divided into six main groups: “*Regosols and Leptosols*”, “*Vertisols*”, “*Cambisols*”, “*Acrisols*”, “*Ferrasols and Plinthosols*”, and “*Fluvisols*”. These 6 groups are sub-divided into 4, 2, 5, 2, 4, and 3 categories of soils, respectively, for a total of 20 categories. This 20-category classification has been established according to the *World Reference Base for Soil Resources* [36] and adapted to the specificities of New Caledonian soils [37].

For the land use variable, three levels of classification were available: the first, second, and third levels consist of 5, 22, and 26 categories, respectively. These three levels of classification corresponds to the level of precision in the land use classification. For example, in the first level, we have the “*artificial lands*” category. Then, in the second level, the “*artificial lands*” category is divided into several categories, e.g., “*urbanized areas*”, “*industrial or commercial zones and facilities*”, and “*artificial green spaces and facilities*”. Finally, in the third level, the “*urbanized areas*” category is once again, divided into more sub-categories, such as “*isolated dwelling*” and “*discontinuous urban fabric*”. In this study, we employed the 22-category division (the second one), as it included the category “*Landfills, construction sites, material extraction*”, which may provide indirect information about the presence of rodents, one of the primary reservoirs of pathogenic *Leptospira* [38]. The remaining categories are the following: “*Urbanized areas*”, “*Structured and managed pastoral lands—meadows*”, “*Arable lands*”, “*Industrial or commercial zones and facilities*”, “*Mines, mining landfills, mining infrastructure and sites*”, “*Wooded areas*”, “*Artificial green spaces and facilities*”, “*Permanent crops*”, “*Tree plantation—forestry*”, “*Communication networks*”, “*Shrub and/or herbaceous formations*”, “*Open spaces, with little or no vegetation*”, “*Maritime wetlands*”, “*Structured and managed pastoral lands*”, “*Inland wetlands*”, “*Continental waters*”, “*Marine waters*”, “*Arable lands and permanent crops*”, “*Tree canopy*”, “*Shrub and herbaceous layers*”, and “*Orchards and small fruits*”.

To integrate soil types (*ST*) and land use (*LU*) variables into the analysis, we calculated the percentage of coverage of a given IRIS by each category of these variables. More specifically, soil types and land use variables are represented by polygons, denoted as *ST* = {*ST*^(1)^, *ST*^(2)^, …, *ST*^(20)^} and *LU* = {*LU*^(1)^, *LU*^(2)^, …, *LU*^(22)^}, respectively. For each IRIS *x*_*i*_, we calculated the proportion of its surface area covered by soil types and land use categories using *I*_*ST*_(*x*_*i*_) and *I*_*LU*_(*x*_*i*_) functions, respectively, defined as:

$$\begin{array}{c} I_{ST}\left(x_{i}\right)=\{\frac{A(ST^{\left(c\right)}\cap x_{i})}{A(x_{i})},\;c\in \left\{1,\cdots ,20\right\}\} \end{array}$$ (1)

$$\begin{array}{c} I_{LU}\left(x_{i}\right)=\{\frac{A(LU^{\left(c\right)}\cap x_{i})}{A(x_{i})},\;c\in \left\{1,\cdots ,22\right\}\} \end{array}$$ (2)

where *A*(⋅) represents the area function, providing the surface area of a given geographic region. Similarly, for the farming area (*FA*) variable represented by a polygon *F*, we applied the same approach with a function *I*_*F*_(*x*_*i*_) defined as:

$$\begin{array}{c} I_{F}(x_{i})=\frac{A(F\cap x_{i})}{A(x_{i})} \end{array}$$ (3)

The altitude variable, represented by a raster map with a resolution of 10 meters, was added to the analysis using the same masking technique used for the meteorological variables, and aggregated by computing the mean, median, and variance within each IRIS.

Finally, we included the number of forest fires identified through point data. A forest fire has several impacts on soils, such as reduction in organic matter, leading to potential soil fertility loss, which affects the presence and survival of *Leptospira*. The Environmental Observatory of New Caledonia has implemented a real-time process utilizing satellite imagery, specifically MODIS and NPP data, to detect forest fires. To integrate this information into our analysis, we tallied the occurrences of point data associated with forest fires within each IRIS. This counting procedure was carried out monthly, covering the period from 2011 to 2022.

#### Demographic variables

In New Caledonia, since the population lives according to three different lifestyles: urban, rural and tribal; we added into our analysis several demographic indicators that may influence the *Leptospira* contamination risk. As the bacteria survive in water and soils, we added variables from population census where inhabitants may interact (directly or undirectly) with the environment. Demographic factors were provided by the Institute of Statistics and Economic Studies (ISEE) of New Caledonia. Besides the population and population density which are common demographic indicators [10], as the tribal lifestyle represents 20% of the population [19], we added the following variables: the “number of people living in tribes”, and the “number of stay-at-home people”. Then, as a third of the population works outside their city of residence [39], we aimed to examine whether the fact that IRIS residents that work outside of their home areas affects the contamination, which led us to include the variable “number of people working in a city other than their home city”. Additionally, we aimed to investigate whether residents’ commuting patterns between their homes, workplaces, and other activities affect *Leptospira* contamination. Therefore, we included the following variables: “number of people whose main mode of transportation is walking”, and “number of people whose main mode of transportation is public transit”. Finally, several works demonstrated the contribution of poverty indicator in the leptospirosis prevalence [40, 41]. Since wealth in New Caledonia is highly heterogeneous [42], we included the following variables as indicators of poverty in a given IRIS: the “number of people working in agriculture”, the “number of households without individual access to water”, the “number of households without access to electricity”, the “number of old houses” (where a house is considered old if it was built at least 10 years before the computed incidence rate), and the “number of people in the working class”.

Although these variables are already organized by IRIS unit, they were collected during population censuses, which happens once every five years i.e., 2009, 2014, and 2019. To prevent the repetition of values for each of these variables over five consecutive years, we applied a simple affine function to estimate them between two population censuses. The estimated values were calculated as follows: once we computed the incidence rate for a month in a given year *y*, if *y* falls between two population censuses (*y*_*i*_, *y*_*j*_), the estimation of the demographic value *v* is defined by linear interpolation: *v*(*y*) = *a* × *y* + *b*, where $a=\frac{y_{j}-y_{i}}{v_{j}-v_{i}}$ and *b* = *y*_*i*_ − *a* × *v*_*i*_. Here, *v*_*i*_ and *v*_*j*_ represent the demographic data corresponding to the population censuses *y*_*i*_ and *y*_*j*_, respectively. For the monthly computed incidence rates of each IRIS from 2019 to 2022, we retained the demographic variables at the same values as those obtained during the most recent population census in 2019.

### Ensemble learning

The risk mapping of leptospirosis has been established using an ensemble learning approach, including four machine learning models: SVM (Support Vector Machine), RF (Random Forest), XGB (Extreme Gradient Boosting), and LR (Logistic Regression). To prepare the dataset, the data was initially normalized using a Min-Max scaler defined by:

$$\begin{array}{c} \text{Min-Max}\left(x_{i,j}\right)=\frac{x_{i,j}-\text{min}\;\left(x_{\cdot ,j}\right)}{\text{max}\;\left(x_{\cdot ,j}\right)-\text{min}\;\left(x_{\cdot ,j}\right)} \end{array}$$ (4)

In this equation, *x*_*i*,*j*_ corresponds to the value associated to the variable *j* for a given IRIS *x*_*i*_, and *x*_⋅,*j*_ is the vector of every IRIS values for the variable *j* in the dataset. Subsequently, the dataset was split into a training set, consisting of data from 2011 to 2020, and a test set, comprising data from 2021 and 2022.

The 80:20 split is commonly used in machine learning where 80% of the initial dataset represent the training set and the remaining 20% represent the test set, ensuring that the month-IRIS composing the test set are completely independent from the month-IRIS composing the training set. However, we set the test set ratio considering a temporal constraint, with 2,736 out of 14,416 month-IRIS (month-IRIS from 2021 and 2022), in order to visualize spatially and temporally the distribution of the contamination risk in New Caledonia.

Then to mitigate the risk of overfitting arising from the imbalanced dataset, the training set, composed of 13,680 month-IRIS, was undersampled. Several studies have attempted to address this issue, notably using the Maximum Entropy (MaxEnt) method [10, 23, 24]. For areas where no cases have been registered, MaxEnt method enables random generation of zones with zero leptospirosis cases. In our approach, we applied an under-sampling approach, in order to include both areas with registered cases and areas with no recorded cases. Indeed, during the period spanning 2011 to 2020, only 573 out of 13,680 month-IRIS had an incidence rate greater than 0. To address this imbalance and ensure a well-distributed training set, we randomly sampled 573 month-IRIS from the remaining 13,107, each with a null incidence rate. This process yielded a balanced dataset comprising 1,146 homogeneous month-IRIS. To guarantee representative outcomes in the ensemble fitting process, we repeated this under-sampling procedure 50 times, resulting in the creation of 50 balanced training sets, each comprised of different null incidence rate month-IRIS. Consequently, a total of 50 ensemble training runs were conducted.

For every run, we fine-tuned each machine learning model using a Grid Search technique coupled with 5-fold cross-validation, which resulted in a 80% for the training set and 20% for validation set.

Throughout this study, our primary focus was on maximizing both the sensitivity and specificity metrics, defined as:

$$\begin{array}{c} \text{Sensitivity}=\frac{\text{TP}}{\text{TP}+\text{FN}}\;True\;Positive\;Rate \end{array}$$ (5)

$$\begin{array}{c} \text{Specificity}=\frac{\text{TN}}{\text{TN}+\text{FP}}\;True\;Negative\;Rate \end{array}$$ (6)

where TP, TN, FP and FN are the true positive, true negative, false positive and false negative, respectively. In this case, the true positive, true negative, false positive and false negative correspond to areas correctly predicted as “presence of risk”, areas correctly predicted as “absence of risk”, areas incorrectly predicted as “presence of risk” and areas incorrectly predicted as “absence of risk”, respectively. Consequently, the Grid Search was fine-tuned to optimize the balanced accuracy metric, which is calculated as:

$$\begin{array}{c} Bal\;Acc=\frac{1}{2}\times (\text{Sensitivity}+\text{Specificity}) \end{array}$$ (7)

In addition to the balanced accuracy metric, we calculated the overall accuracy to assess how closely the predictions align with the actual response (absence/presence of risk). The accuracy is defined as:

$$\begin{array}{c} Acc=\frac{\text{TP}+\text{TN}}{\text{TP}+\text{TN}+\text{FP}+\text{FN}} \end{array}$$ (8)

Then, we performed the concordance statistic (*c*-statistic) on our predictions as measure of discrimination. It is also known as the area under the ROC curve (AUC-ROC) which is a commonly used metric [43]. This metric allowed us to evaluate the discrimination ability in our predictions. In addition, as measure of calibration, we performed the Brier loss score which is commonly used as well [44]. This loss score measures the mean squared difference between the predicted probabilities and the actual binary outcomes. The closer the Brier loss is to 0, the better the model is considered to be calibrated.

Upon completing the 50 ensemble training iterations, each involving four machine learning models, we obtained a total of 200 fine-tuned machine learning models. However, to speedup prediction time, we only retained the top 5% of the models based on balanced accuracy Eq (7). This resulted in selecting 10 models for prediction on the test set. There was no heterogeneity in estimates of model parameter values and performance to handle. In the ensemble learning approach, each machine learning model was adjusted using a grid search strategy, i.e., we provided a wide range of parameter that we varied. Then, with the grid search approach, we retained the parameters of each machine learning model that result in the best performance, i.e., the balanced accuracy. Additionally, we save the specificity and sensitivity metrics of each selected model. Those metrics are then used to weight their predictions during the testing phase. Given a presence/absence of risk associated to a month-IRIS *x*_*i*_ predicted by *n* models, the weighted prediction *P*(*x*_*i*_) is defined by:

$$\begin{array}{c} P(x_{i})=\underset{p\;\in \;\{A,B\}}{\text{max}}\;\sigma \left(p\right) \end{array}$$ (9)

$$\begin{array}{c} A=\frac{2}{n}\times \sum_{j=1}^{n}{\text{Specificity}}_{j}\times |p_{j}\left(x_{i}\right)-0.5|\times 1_{p_{j}\left(x_{i}\right)\;<\;0.5} \end{array}$$ (10)

$$\begin{array}{c} B=\frac{2}{n}\times \sum_{j=1}^{n}{\text{Sensitivity}}_{j}\times |p_{j}\left(x_{i}\right)-0.5|\times 1_{p_{j}\left(x_{i}\right)\;\geq \;0.5} \end{array}$$ (11)

In these equations, the sum iterates *j* over the *n* models. Finally, $1_{X}$ is the indicator function equals to 1 if the criterion is satisfied and to 0 otherwise.

To summarize, if a model predicts an “absence of risk” (i.e., the predicted probability is lower than 0.5), it has been weighted based on its specificity Eq (10). Conversely, if it predicts a “presence of risk” (i.e., the predicted probability is equal to or greater than 0.5), it has been weighted according to its sensitivity Eq (11). Additionally, to ensure that the probabilities sum up to 1, we applied a softmax function Eq (12), denoted by *σ*(⋅) and defined by:

$$\begin{array}{c} \sigma {\left(z\right)}_{j}=\frac{e^{z_{j}}}{\sum_{k=1}^{K}e^{z_{k}}},\;\forall \;j\in \left\{1,...,K\right\}\;\text{and}\;z=\left(z_{1},...,z_{K}\right)\in R^{K} \end{array}$$ (12)

Where *K* corresponds to the number of classes available in the dataset. In this case, we have two classes (i.e., “absence of risk” and “presence of risk”).

The entire ensemble learning process was conducted using the Python language (version 3.8.13).

### Importance variable identification

To provide further insights into the results, we assessed the importance of the input variables in predicting the risk of *Leptospira* contamination using a permutation technique. Numerous studies have aimed to identify the most important variables contributing to the leptospirosis distribution, such as the permutation technique coupled with partial dependence plots [14] and the jackknife test [10, 23]. In this paper, we computed the important variable identification using a hierarchical clustering coupled with group permutations. The computation is applied as follows:

1. The balanced accuracy Eq (7) from the weighted prediction is computed on the test set and serves as a baseline performance metric denoted *Bal Acc*_*raw*_.
2. An ascending hierarchical clustering is applied to handle multicollinear or correlated variables. The clustering is performed using Ward’s linkage method with a fixed cutoff (*t* = 1.075) to obtain the clustered variables.
3. Within each group, the input values are randomly shuffled. This shuffling breaks any inherent relationship between the variables while preserving the distribution of values within each variable.
4. The balanced accuracy (*Bal Acc*_*perm*_) is computed on the shuffled data.
5. The absolute percent error (Δ_*v*_) is calculated using the following formula:
  $$\begin{array}{c} \Delta_{v}=100\times |\frac{Bal\;Acc_{perm}-Bal\;Acc_{raw}}{Bal\;Acc_{raw}}| \end{array}$$ (13)
  The higher the Δ_*v*_, the more significant its corresponding group variable.

In order to obtain representative results, we repeated the random permutation 2,500 times from the third step.

Regarding the fixed cutoff value, it was initially determined visually. Although there are several approaches to determine the optimal cutoff, such as the scree plot, the silhouette index and intra-cluster inertia, we chose to set the cutoff so that each cluster would be composed of at least three variables. This choice was made because we aimed to determine whether it was possible to separate meteorological variables, particularly accumulated rainfall with one, two, and three-months lags, each described by three variables, i.e., the average, median, and variance of accumulated rainfall. This separation would allow us to more precisely determine the contribution of the accumulated rainfall in different months prior to the computed incidence rate.

Although we experimented the rest of the analysis with higher cutoffs, this resulted in a lower number of final clusters. However, because more variables were grouped within the same clusters, we were unable to precisely determine using the permutation technique, which type of variables (meteorological, demographic, environmental, etc.) contributed the most to the risk of contamination.

To evaluate the most important clusters in the predictions, we selected those with the highest contributions according to the balanced accuracy, i.e., the model’s capability to predict both actual “presence of risk” and “absence of risk”. Therefore, to determine the number of clusters that contribute the most to the predictions, we used two methods.

The first one is by displaying the scree plot of the median of variation in balanced accuracy. Using the scree plot and elbow rule, we were able to identify the clusters that contribute the most to the predictions.

The second method is used to confirm the scree plot. To do so, we performed pairwised statistical tests following 3 steps.

1. First, we assessed whether the data in each sample are normally distributed using Shapiro-Wilk test.
2. Second, we performed Levene’s test to check if the variances of two samples are equal.
3. If both samples are normally distributed and have equal variances, we used the two-sample t-test to verify if one sample mean is significantly greater than the other; Otherwise, we used the Mann-Whitney U Test to verify whether the distribution of one sample is significantly greater than the other.

This second method allowed us to confirm whether the clusters selected from the scree plot are statistically greater than the other clusters according to the variation in the predictions obtained with the group permutations.

Finally, to monitor how our model responds as the variables vary within the most important clusters, we proceeded with an increment technique as outlined below:

For each cluster obtained from the ascending hierarchical clustering:

1. We applied an increment defined by *v*′ = *v* + *v* × *x* for each variable *v* contained within the cluster and for *x* ∈ {−1, −0.9, −0.8, −0.7, …, 0.8, 0.9, 1.0}.
2. Since our data has been normalized using a Min-Max scaler Eq (4), we clipped the incremented values *v*′ between 0 and 1.
3. The ensemble prediction is performed on the new values *v*′.

This method allowed us to observe whether the risk of *Leptospira* contamination increases or decreases with variations in our most significant variables.

## Results

### Spatio-temporal distribution of leptospirosis in New Caledonia

**Fig 4:**
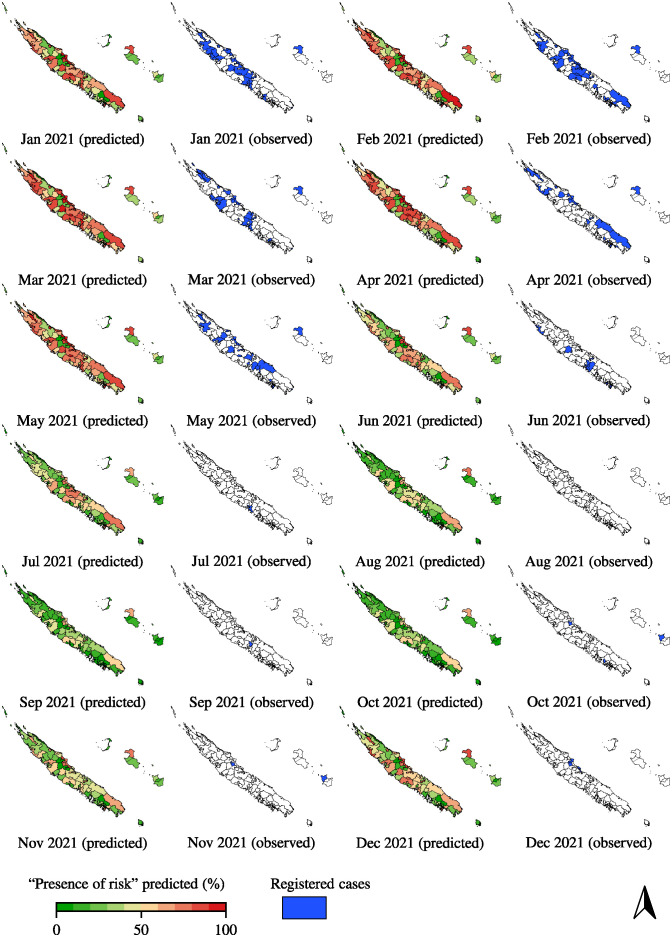
Predicted risk on months of 2021. The risks were obtained from the probability of the “presence of risk” predicted during the weighted ensemble prediction Eq (9). In addition, a Min-Max scaler has been applied in the weighted prediction to range the probability between 0 and 1. The IRIS border shapes were provided by the Institute of Statistics and Economic Studies of New Caledonia (https://ncl.popgis.spc.int/).

From the ensemble learning approach involving 200 machine learning models, we kept the best 5% of models (i.e., 10 models) based on the balanced accuracy Eq (7). All results from each retained machine learning model are detailed in S1 Table. On our 2,736 month-IRIS (114 IRIS × 12 months × 2 years) from the test set (2021–2022), the weighted prediction resulted in a sensitivity of 83.93%, a specificity of 68.46% and a balanced accuracy of 76.19%. The predictions performed a *c*-statistic (AUC-ROC) of 83.29% which indicates a good discrimination ability and a strong model, and an accuracy of 70.36%. In addition, based on our predictions and the actual absence/presence of risk contamination, we obtained a Brier loss score of 0.2 which indicates a well calibrated model with probabilities that are reasonably accurate. The table of training/validation and test sets with and without leptospirosis cases is detailed in the S2 Table. The Fig 4 illustrates the probability Eq (9) of the predicted “presence of risk” for month-IRIS data in 2021 generated by our ensemble learning approach. As depicted in the Fig 4, the risk of *Leptospira* contamination exhibited a seasonal trend, with higher predicted risk levels observed during the warm and rainy season i.e., at the beginning of the year. Conversely, during the cold and dry season, particularly in August, September, and October, the “presence of risk” predicted tends to be lower. In the IRIS of *Sarraméa* (Fig 1) which recorded the highest incidence rate (93.98 cases per 10, 000 inhabitants in January 2021), our ensemble approach predicted a “presence of risk” of 85.98%. Similarly, in three other IRIS areas—*Goyetta*, *Bondé*, and *Unia* (Fig 1)—which also reported high monthly incidence rates (69.24 cases per 10,000 inhabitants in January 2021, 61.35 cases per 10,000 inhabitants in April 2021, and 43.73 cases per 10,000 inhabitants also in April 2021), our ensemble approach predicted “presence of risk” of 87.14% in January 2021, 94.19% in April 2021, and 80.42% in April 2021, respectively.

In 2022 (S1 Fig), the predicted “presence of risk” remains consistently high throughout the year. This forecast is not unexpected, given that the country experienced its highest number of leptospirosis cases in our period analysis (Fig 2). New Caledonia’s median of monthly accumulated rainfall was 80.1 *mm* during the 2011–2020 period, this median being significantly increased to 153.95 *mm* during the 2021–2022 period which increased the risk of contamination. This difference caused by La Niña resulted in a consistently high predicted “presence of risk”, especially in 2022 (Fig 4 and S1 Fig) with a median “presence of risk” predicted of 0.43 (with an interquartile range of 0.48) while in 2021, the median “presence of risk” predicted is of 0.35 (with an interquartile range of 0.44).

Overall, our approach well predicted the seasonal pattern, as it determined a median presence of risk of 0.31 during the cold and dry season (May to October) and 0.47 during the warm and rainy season (November to April) in the test set (month-IRIS of 2021 and 2022), with an interquartile range of 0.40 and 0.48, respectively. These relatively high interquartile ranges suggest that there is a high dispersion in the predicted risk of contamination. Although, it also indicates that certain areas in New Caledonia are likely at significantly higher risk than others, which could warrant more targeted and specific prevention.

### Factor identification in the risk distribution

**Fig 5:**
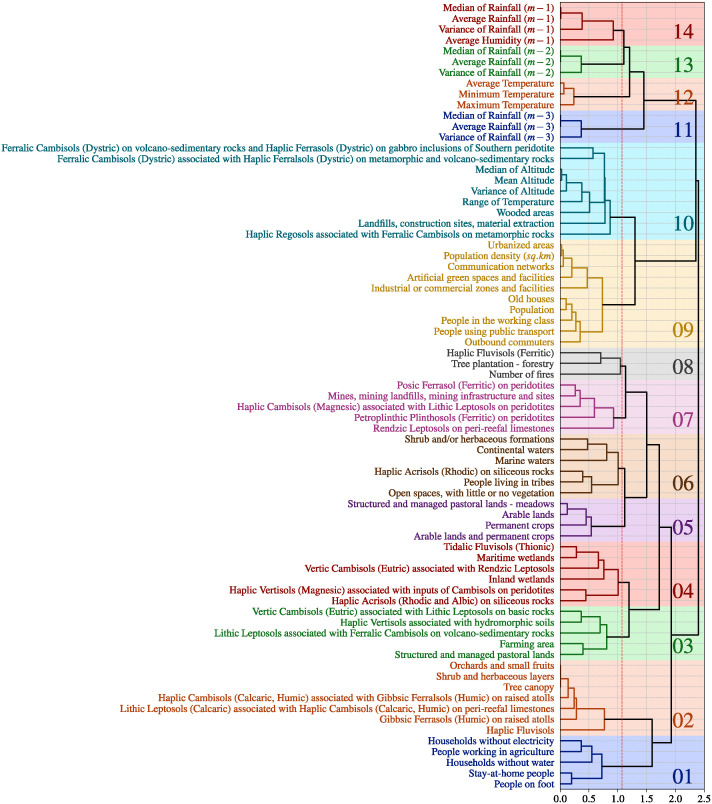
Clusters obtained from the ascending hierarchical clustering. The clustering has been obtained using Ward’s linkage method with *t* = 1.075 as cutoff (red dashed line) and resulted in 14 clusters.

Following the ensemble prediction results, we used a permutation technique to determine the most influential factors. Due to collinearity among various factors, we conducted an ascending hierarchical clustering to group collinear or correlated variables together. Using Ward’s linkage method with a cutoff of *t* = 1.075, we generated a dendrogram depicting 14 clusters (Fig 5). The clustering process effectively grouped the climatic variables by month (clusters 11, 12, 13 and 14) as well as demographic variables (clusters 01 and 09), as illustrated in the Fig 5.

**Fig 6:**
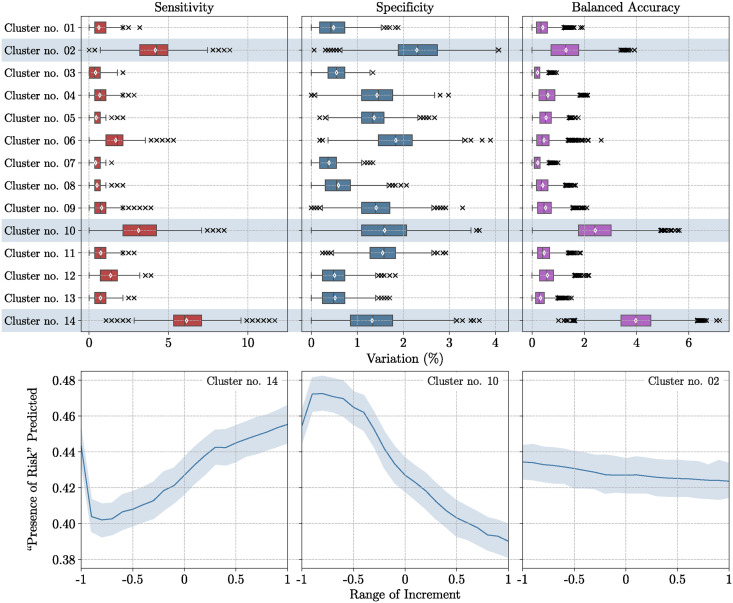
Variable Importance (%) calculated (top panel) and ensemble predictions according to increment technique (bottom panel). The importance score Eq (13) has been computed from 2,500 permutations in the ensemble approach. The increment technique has been applied on the three most important clusters.

The Fig 6 displays in the top panel the percentage error (Δ_*v*_) resulting from the permutation of variables at the scale of each cluster, assessing the impact of variable permutations within individual clusters on the overall ensemble prediction. Additionally, the bottom panel of the Fig 6 displays the predicted “presence of risk” according to the increments applied on the most important clusters.

To determine which clusters contribute the most to the predictions, we displayed the scree plot of the median of variation in balanced accuracy (S2 Fig). Using elbow rule, the clusters 14, 10, and 02 appeared to stand out of the rest of the clusters.

Then, to statistically confirm the scree plot, we performed pairwised statistical tests, detailed in the Importance variable identification subsection. Based on Shapiro-Wilk and Levene tests, none of the clusters are normally distributed. Thus, we performed pairwised one-sided Mann-Whitney U Test to check whether the distribution of balanced accuracy variations from permutations of a cluster is significantly greater than each of every other clusters’ distribution. In this case, the distribution of a cluster corresponds to the different variations in balanced accuracy obtained from group permutations of variables within the associated cluster. In the S3 Table, each *p*-value corresponds to the Mann-Whitney U test with the following hypothesis:

- $H_{0}:C_{i}=C_{j}\;\text{and}\;H_{1}:C_{i}<C_{j}$

where *C*_*i*_ is the distribution of the cluster in row and *C*_*j*_ is the distribution of the cluster in column.

From all the *p*-values, the clusters 14, 10, and 02 appeared to be significantly greater than the rest of the clusters. Moreover, the fourth most contributing cluster according to the scree plot, i.e., the cluster 12, did not appear to be signicantly greater than the rest of the clusters as it resulted in no significant difference with the cluster 04.

As depicted in the scree plot (S2 Fig), in the statistical tests (S3 Table), and in the top panel of the Fig 6, the most critical variables in the ensemble prediction belong to cluster 14, associated with rainfall and humidity from the previous month (*m* − 1). The permutations within this group caused an average variation of 4% in the balanced accuracy. In its bottom panel, the Fig 6 demonstrates that as the values within the cluster 14 increase, the predicted “presence of risk” increases as well.

Subsequently, the two other notable clusters in the ensemble prediction, include clusters 02 and 10. As illustrated in the Fig 6, when the values within the cluster 10 increase, the predicted “presence of risk” decreases. These clusters are linked to soil types, temperature, altitude, and a specific land use category, namely, the “*Landfills, construction sites, material extraction*” category. In particular, the soil types in these clusters correspond mostly to Ferralic, Cambisols and Regosols, which are known to be rich in organic matter [37].

## Discussion

In this study, we established the first precise spatio-temporal risk mapping of leptospirosis at the finest possible spatio-temporal scale, integrating various factors (meteorology, topography, socio-demographic). The present study identified the seasonal pattern of leptospirosis outbreaks, which occur during the warm and rainy period (November to April). In addition, based on the grouped permutation technique, our study demonstrated that rainfall and humidity with a 1-month lag contribute the most to the risk of contamination, with the “presence of risk” prediction increasing as the values within the cluster 14 increase. The first finding can be explained by heavy rainfall promoting *Leptospira* resuspension [6] as the bacteria can survive for months in the environment [3], leading to leptospirosis outbreaks [18]. This result has also been identified at the national level as well as in other tropical regions [45]. The second finding appears to confirm the first result, demonstrating that as cumulative rainfall and humidity intensify, the predicted “presence of risk” increases. This result is not unexpected, as it has been proven in other tropical regions [46, 47]. With the climate change, New Caledonia is confronted to more frequent heavy rainfall [48]. Along with the seasonal pattern detected by our approach, it has also considered the long period of La Niña that occurred in 2021 and 2022 [49] with 22 months out of 24 indicating La Niña episode, resulting in a high “presence of risk” detected. In opposite to El Niño, La Niña is a climate phenomenon in the Pacific Ocean characterized by a cooler-than-average sea surface temperature. This shift alters weather patterns across the region, typically bringing wetter conditions and increased tropical cyclone activities to the western Pacific. In New Caledonia, La Niña often results in heavier rainfall and a higher likelihood of cyclones, making the weather more unpredictable and stormier than usual.

Besides rainfall and humidity from the previous month that contribute the most to the risk of contamination, other factors have to come into consideration. Our present results demonstrated that in high-altitude, the “presence of risk” prediction decreases. This result indicates that high-altitude areas characterized by a higher slope and less stagnant water, are less prone to flooding, making the transmission of *Leptospira* more difficult. Conversely, in low-altitude regions where there is a high concentration of the population, the likelihood of flooding and risk contamination are also higher [52, 53].

One notable factor that comes into consideration is the soil types covering New Caledonia. Indeed, the soil types within the clusters 02 and 10 are known to be rich in organic matter [37] and *Leptospira* is commonly associated with soils that have high organic matter content [54, 55]. However, it is important to recall that nature operates according to a complex balance. Thus, an excess of organic matter is not necessarily beneficial: As depicted on the bottom panel of the Fig 6, a high coverage of soil rich in organic matter can also negatively affect the presence and survival of *Leptospira*, leading to a decreased risk of *Leptospira* contamination. An excess of organic matter can lead to more intense anaerobic decomposition which may reduce oxygen availability in the soil, as the bacteria require aerobic conditions for survival [56].

Several studies in Nicaragua [57]; in Fiji [58] and in Brazil [59] have revealed the impact of demographic variables in the *Leptospira* contamination. In New Caledonia, despite its small surface area (18,576 *km*^2^) and the tribal lifestyle representing 20% of the population [19], the integrated demographic variables such as the number of people living in tribes and the number of people working in agriculture, contained within clusters 01, 06, and 09, do not appear to significantly contribute to the contamination risk of *Leptospira* with an average variation lower than 1% in the balanced accuracy. This low contribution may particularly be due to the fact that, given its small surface area, inhabitants in New Caledonia are eager to move around the country more frequently. Indeed, hunting and fishing, which require to move from one area to another, are deeply rooted in the culture. Beyond being leisure activities, they hold a significant cultural role for most of the country’s inhabitants [37, 60, 61]. Additionally, the population appears to be highly mobile, with inhabitants frequently traveling long distances for work or to reunite with family [62]. Thus, the risk of *Leptospira* contamination may not be related to inhabitants living within a specific IRIS, but rather to the environment and ecosystem of that IRIS [63], which constitutes a confounding factor.

Although, it is important to recall that these group contributions are relative to each other. Therefore, some groups of variables, typically clusters associated to demographic variables, may still contribute to the *Leptospira* contamination as has been observed in Brazil [64]. However, in New Caledonia, based on the population censuses that take place once every five years, demographic variables do not appear to be as contributing as the meteorological variables.

The low influence of demographic data can also be attributed to the fact that *Nouméa* and its peripheral cities represent 67.2% of the total population [25], which corresponds to only 39 out of the 114 IRIS. The remaining population, which represents a significantly smaller percentage, is spread across the other 75 IRIS, which can lead to IRIS with zero inhabitants. As a result, many IRIS had few or no inhabitants, which likely impacted the analysis.

Although our approach presented satisfying results with the seasonal pattern predicted, we can identify some uncertainty that have impacted our spatio-temporal analysis. Despite the surveillance data consolidation since 2011 conducted by the Health and Social Affairs Department of New Caledonia to obtain the most precise contamination localization possible, it remains difficult to determine precisely where the infection may have occurred. With 35% of the population residing in the capital, *Nouméa*, and a total country surface area of 18,576 *km*^2^ [25], inhabitants tends to move more frequently around the country, which may complicate the precise localization of infectious hotspots.

In addition, some of our integrated variables do not have the temporal dynamics, in particular the land use variable. The results could be improved by using alternative maps that include the temporal dynamics. The land use classification we have taken into account has been established by the Government of New Caledonia and the Environment Observatory of New Caledonia using SPOT6 satellite imagery. Recent works have demonstrated the capability of deep learning techniques for land cover and land use classification using the same satellite images [65]. By using the different spectral bands available in satellite imagery from several sources (SPOT6, Sentinel, Landsat), it would be possible to obtain the land use evolution on a monthly scale. In their work [65], the authors also published their training data [66]. Although, the training data represents 5 areas of New Caledonia for a total surface area of 128.4 *km*^2^. As stated in their study, additional information and work are necessary to cover the specific conditions in the rest of the country.

While the significance of spatial and temporal scales has been demonstrated [67, 68], conducting a risk mapping of leptospirosis on a monthly scale with IRIS precision presented some limitations. As illustrated in the Fig 2, there are specific months where dividing the total number of leptospirosis cases in a given month by the 114 IRIS units resulted in a majority of IRIS units having a null incidence rate, thereby creating a highly imbalanced dataset. In regions where leptospirosis cases have been registered, there is a certainty about the risk of infection; however, in regions where no cases have been detected during a given period, this does not necessarily indicate an absence of contamination risk. Ultimately, despite the under-sampling technique employed to mitigate the imbalanced dataset, that bias is reflected by our moderate specificity of 68.46%.

Although the spatio-temporal analysis has been applied to *Leptospira* contamination cases, these results highlighted the potential for applying this analysis to other neglected zoonosis and waterborne diseases occurring in various climates, such as leishmaniasis [50] and anthrax [51].

## Conclusion

In summary, we conducted a spatio-temporal analysis of the presence of *Leptospira* contamination risk in New Caledonia over a 12-year period (2011–2022). To determine the distribution of leptospirosis risk during this time-frame, we integrated a large number of factors (meteorologic, environmental and demographic). In addition, the analysis has been conducted using several metrics (mean, median and variance) on our factors. Finally, we identified the accumulated rainfall and humidity with a 1-month lag, the soil type and the altitude as the most important factors influencing the risk distribution of leptospirosis. The results could be improved by considering soil factors, such as pH, salinity and other soil physico-chemical parameters.

The study’s findings have significant applicability for public health efforts aimed at controlling and preventing leptospirosis. By providing precise spatio-temporal risk mapping and identifying key environmental and meteorological factors, the results can be used to enhance disease surveillance systems, allowing for early detection and timely response to outbreaks.

The methodology developed can be adapted and applied to other geographic areas facing similar environmental challenges, making the approach broadly generalizable beyond the study region. This flexibility enables public health authorities in various settings to tailor interventions based on localized risk factors, optimize resource allocation, and design targeted prevention strategies.

Ultimately, the study provided a robust framework that can be utilized to mitigate the leptospirosis burden and other neglected climate-sensitive zoonotic diseases, such as leishmaniasis or anthrax.

## Supporting information

S1 Fig Predicted risk on months of 2022. The risks were obtained from the probability of the “presence of risk” predicted during the weighted ensemble prediction Eq (9). The IRIS border shapes were provided by the Institute of Statistics and Economic Studies of New Caledonia (https://ncl.popgis.spc.int/).
(PDF)

S2 Fig Scree plot of the median of variation in balanced accuracy from group permutations. Using elbow rule, clusters 14, 10, and 02 appeared to stand out of the rest of the clusters.
(PDF)

S1 Table Metrics (%) computed on the test set. The test set concerns month-IRIS of 2021 and 2022.
(PDF)

S2 Table Number of month-IRIS with and without leptospirosis cases. The table is divided into training/validation and test sets. Additionally, the numbers represent the count of month-IRIS between 2021 and 2022 that recorded either zero or at least one case of leptospirosis.
(PDF)

S3 TableTable of *p*-values from one-sided Mann-Whitney U Test.Red cells correspond to *p*-values < 0.05, and therefore the $H_{0}\;:\;C_{i}=C_{j}$ with *C*_*i*_ the cluster in row and *C*_*j*_ the cluster in column is rejected.(PDF)
