# Supplementary figures and images for: Spatio-temporal risk prediction of leptospirosis: A machine-learning-based approach
Source: PLoS Negl Trop Dis. 2025 Jan 16;19(1):e0012755. doi: 10.1371/journal.pntd.0012755 (PMC11737754; doi:10.1371/journal.pntd.0012755)

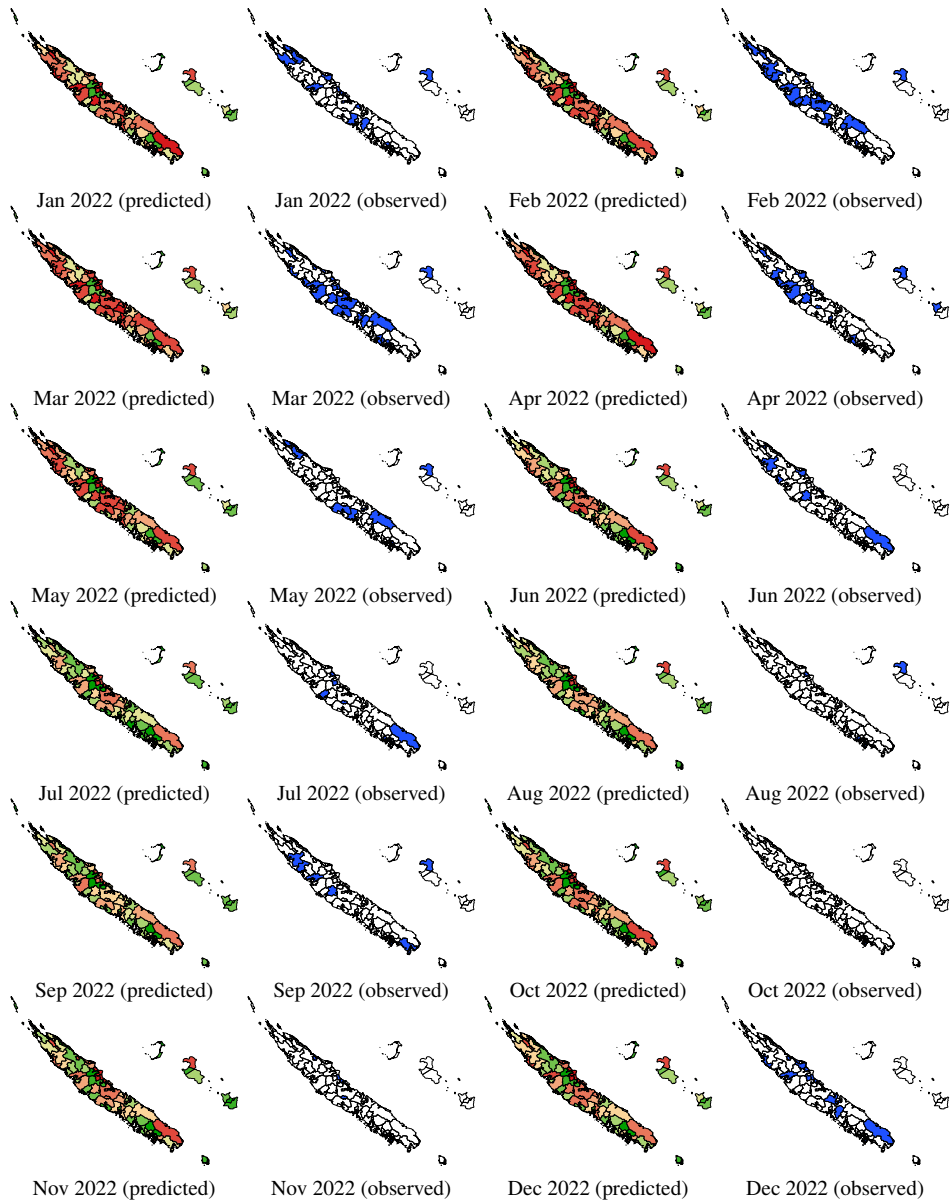

“Presence of risk” predicted (%)

Registered cases

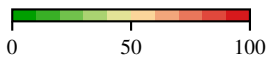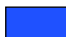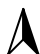

Supplement: S1 Fig — The risks were obtained from the probability of the “presence of risk” predicted during the weighted ensemble prediction Eq (9). The IRIS border shapes were provided by the Institute of Statistics and Economic Studies of New Caledonia (https://ncl.popgis.spc.int/). (PDF) [file pntd.0012755.s001.pdf]

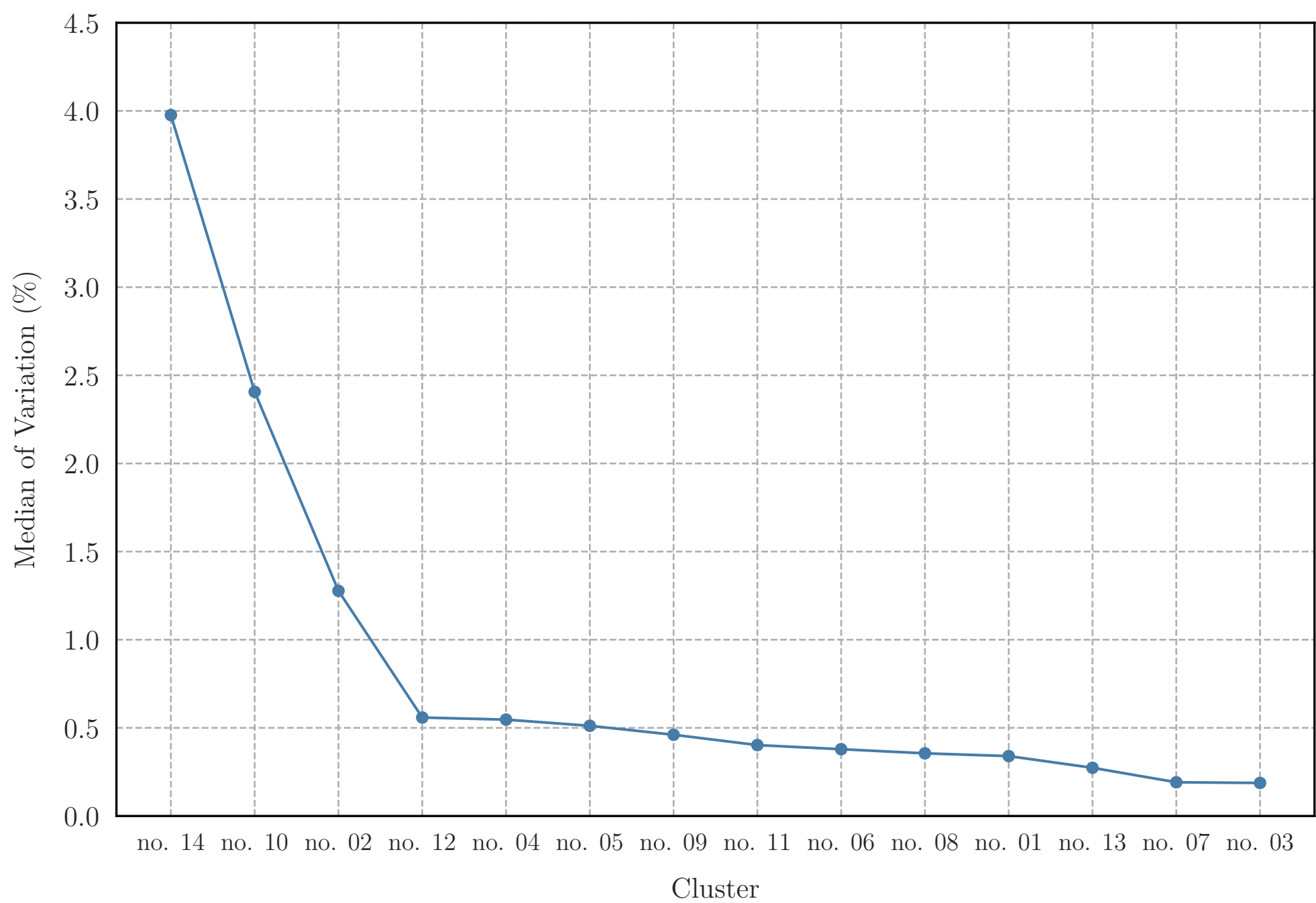

Supplement: S2 Fig — Using elbow rule, clusters 14, 10, and 02 appeared to stand out of the rest of the clusters. (PDF) [file pntd.0012755.s002.pdf]
