# Supplementary material for: Spatio-temporal risk prediction of leptospirosis: A machine-learning-based approach
Source: PLoS Negl Trop Dis. 2025 Jan 16;19(1):e0012755. doi: 10.1371/journal.pntd.0012755 (PMC11737754; doi:10.1371/journal.pntd.0012755)
Supplement: S1 Table — The test set concerns month-IRIS of 2021 and 2022. (PDF) [file pntd.0012755.s003.pdf]

Top	Model	AUC	Accuracy	Recall	Specificity	*Bal Acc*
–	Ensemble	**0.8329**	0.7036	0.8393	0.6846	**0.7619**
1	SVM	0.8097	0.6835	0.8333	0.6625	0.7479
2	RF	0.8176	0.6773	0.8423	0.6542	0.7482
3	SVM	0.816	0.6871	0.8304	0.6671	0.7487
4	SVM	0.8169	**0.7142**	0.7946	**0.7029**	0.7488
5	RF	0.817	0.6817	0.8393	0.6596	0.7494
6	SVM	0.8206	0.6893	0.8304	0.6696	0.75
7	RF	0.8134	0.6988	0.8185	0.6821	0.7503
8	RF	0.8173	0.6795	**0.8482**	0.6558	0.752
9	RF	0.8116	0.6842	0.8452	0.6617	0.7535
10	RF	0.8064	0.6937	0.8333	0.6742	0.7538
