# Supplementary material for: Spatio-temporal risk prediction of leptospirosis: A machine-learning-based approach
Source: PLoS Negl Trop Dis. 2025 Jan 16;19(1):e0012755. doi: 10.1371/journal.pntd.0012755 (PMC11737754; doi:10.1371/journal.pntd.0012755)
Supplement: S2 Table — The table is divided into training/validation and test sets. Additionally, the numbers represent the count of month-IRIS between 2021 and 2022 that recorded either zero or at least one case of leptospirosis. (PDF) [file pntd.0012755.s004.pdf]

Sets	Time frames	IRIS with cases	IRIS without cases	Total
Training/Validation	2011–2020	573	13,107	13,680
Test	2021–2022	336	2,400	2,736
Total	2011–2022	909	15,507	14,416
